# Blood Neutrophil/Lymphocyte Ratio Is Associated With Cerebral Large-Artery Atherosclerosis but Not With Cerebral Small-Vessel Disease

**DOI:** 10.3389/fneur.2020.01022

**Published:** 2020-09-09

**Authors:** Darda Chung, Kee Ook Lee, Jung-Won Choi, Nam Keun Kim, Ok-Joon Kim, Sang-Heum Kim, Seung-Hun Oh, Won Chan Kim

**Affiliations:** ^1^Department of Neurology, CHA Bundang Medical Center, CHA University, Seongnam, South Korea; ^2^Institute for Clinical Research, CHA Bundang Medical Center, CHA University, Seongnam, South Korea; ^3^Department of Radiology, CHA Bundang Medical Center, CHA University, Seongnam, South Korea

**Keywords:** neutrophil-lymphocyte ratio, ischemic stroke, large-artery atherosclerosis, small-vessel disease, cardiovascular risk factors

## Abstract

**Background and Objective:** The blood neutrophil/lymphocyte ratio (NLR) is a marker of peripheral inflammation, with a high NLR associated with an increased risk of cardiovascular events and poor prognosis in ischemic stroke. However, few data are available on the differential impact of the blood NLR on different silent cerebral vascular pathologies, including cerebral large-artery atherosclerosis (LAA) and cerebral small-vessel disease (CSVD), in neurologically healthy individuals.

**Methods:** We evaluated cardiovascular risk factors, brain magnetic resonance imaging (MRI), and MR angiography of 950 individuals without any neurological diseases. The study participants were divided into three groups according to NLR tertile (low, middle, and high). The presences of extracranial (EC) and intracranial (IC) atherosclerosis were considered to indicate LAA on brain MR angiography. The presences of silent lacunar infarction (SLI) and cerebral white matter hyperintensities (WMHs) were analyzed as indices of CSVD on brain MRI.

**Results:** In univariate analysis, the high NLR tertile group showed a high prevalence of old age, hyperlipidemia, and renal dysfunction and higher fasting glucose. Multivariable logistic regression analysis revealed that indices of LAA (EC atherosclerosis [odds ratio: 1.88; 95% confidence interval: 1.14–3.09; *p* = 0.01] and IC atherosclerosis [odds ratio: 1.87; 95% confidence interval: 1.15–3.06; *p* = 0.01]) were more prevalent in the highest NLR tertile group than in the lowest NLR tertile group after adjustment for cardiovascular risk factors. However, no significant differences were found in the prevalence of CSVD indices (SLI and WMHs) among the three NLR tertile groups.

**Conclusions:** A high NLR is associated with the development of cerebral LAA but not CSVD.

## Introduction

Vascular risk factor modification is important for future stroke prevention. Ischemic stroke (IS) is caused by heterogeneous vascular pathologies in the brain (1). One of the major vascular pathologies of IS is large-artery atherosclerosis (LAA) in carotid-vertebral arteries in the extracranial portion (EC atherosclerosis) and in major cerebral arteries in the intracranial portion (IC atherosclerosis). Atherosclerosis develops through multiple cascades of lipid accumulation, endothelial damage, platelet deposition, and smooth muscle cell migration and proliferation (2, 3). The pathophysiology of atherosclerosis is also strongly affected by vascular inflammation (4–7). During plaque formation, an imbalanced inflammatory reaction of the large- and medium-sized arterial wall occurs in response to specific stimuli that produce a wide range of circulating inflammatory molecules. Vascular inflammation promotes the development and progression of atherosclerosis and induces plaque rupture, platelet aggregation, and vascular thrombosis, which lead to increased risk of IS (8). High-sensitivity C-reactive protein (hs-CRP), interleukin (IL)-6, IL-8, interferon-inducible protein of 10 kDa, and monocyte chemoattractant protein (MCP)-1 have been linked to plaque destabilization, microvascular dysfunction, and adverse outcomes of cardiovascular disease and IS (9–12). Recent work revealed that genetically determined circulating MCP-1 was associated with the risk of IS and LAA-type IS, suggesting that inflammation is not merely a bystander in atherosclerosis, but a predisposing factor for LAA development (13). The CANTOS trial (Canakinumab Anti-Inflammatory Thrombosis Outcome Study) demonstrated the potential ability of IL-1β and pro-inflammatory cytokine targeting to reduce vascular end points, including the rate of recurrent cardiovascular events, such as nonfatal myocardial infarction, nonfatal stroke, and cardiovascular mortality, among patients with myocardial infarction and elevated circulating CRP levels (14).

Cerebral small-vessel disease (CSVD), another cerebral vascular pathology, is caused by the occlusion of small perforating arteries or arterioles in the elderly brain (15, 16). On brain imaging, CSVD usually presents as silent lacunar infarction (SLI), cerebral white matter hyperintensities (WMHs), and cerebral microbleeds. CSVD has a distinct pathogenesis from LAA, such as lipohyalinosis, segmental demyelination, and endothelial dysfunction (17). Its development has also been linked to inflammation in recent studies (18–20). In 519 neurologically healthy people, the presence of SLI was associated with an increased serum hs-CRP level (18). In the Atherosclerosis Risk in Communities (ARIC) study, mid-life serum hs-CRP level was associated with unstable white matter integrity and WMH volume in the elderly (20). Although several clinical observations have investigated hs-CRP and other inflammatory cytokines (e.g., IL-6, IL-8, MCP-1) as biomarkers of LAA and/or CSVD, their clinical use is limited due to high variability, a lack of a standardized reference across laboratories, and low cost-effectiveness.

The neutrophil/lymphocyte ratio (NLR) has also been reported as a useful marker of inflammation (21). This ratio is easily obtained from a routine complete blood count. The NLR is attracting attention as a potential biomarker because of its usefulness as a strong prognostic factor in various diseases, including cerebrovascular diseases (22–26). The ratio is considered a better predictor of cardiovascular disease than the total number of white blood cells or the neutrophil count alone (22). In addition, recent studies have shown that the NLR is an independent factor for predicting short-term mortality in patients with IS, particularly in the acute phase (27–29). However, most studies regarding the clinical value of the NLR in IS were performed during the acute phase of IS, which may not reflect its actual effect on atherosclerosis or small-vessel pathology in the silent period before IS. Thus, evaluation of the NLR in the asymptomatic period may clarify the contributing role of inflammation to LAA or CSVD. Furthermore, whether NLR is differentially associated with two distinct silent cerebral vascular pathologies (LAA and CSVD) has not yet been studied in a single cohort. Here, we aimed to determine the value of the NLR as an inflammatory marker and stratify the analysis according to meaningful subgroups defined by atherosclerotic etiology.

## Materials and Methods

### Study Population

This was a hospital-based, cross-sectional study. The study participants were neurologically healthy participants without stroke, aged ≥ 45 years, who visited the outpatient clinic of the Department of Neurology or the healthcare center at CHA Bundang Medical Center, Seongnam, Korea, for routine health examination between March 2008 and December 2014. This study was conducted according to the guidelines of the Declaration of Helsinki and approved by the Institutional Ethical Committee of the CHA Bundang Medical Center (IRB approval no. 2010-083).

All of the participants were neurologically healthy but sought medical attention because they had underlying cardiovascular risk factors or a family history of stroke. We only included individuals who underwent brain magnetic resonance imaging (MRI) and magnetic resonance angiography (MRA). We reviewed the medical records, laboratory test results, and radiological findings of all of the study participants; these data were extracted from a database. We only included patients whose records contained adequate demographic, radiological, and laboratory data. We did not include patients with recent infection history, clinical fever higher than 37.2°C, or any other neurologic disorders. Of 1,011 study patients extracted from our database during the study period, we excluded 61 for the following reasons: (1) inadequate medical information (*n* = 5); (2) no laboratory tests performed (*n* = 12); (3) no data on brain MRI or MRA (*n* = 24); (4) previous history of neurological disease (*n* = 9); or (5) abnormal neurological findings at the time of examination (*n* = 8). A total of 950 participants were included in the subsequent analysis.

### Risk Factor Assessment

We reviewed patients' medical records to gather information on their medical history and laboratory data related to cardiovascular risk factors. Hypertension was defined as a high baseline blood pressure (systolic ≥ 140 mmHg or diastolic ≥ 90 mmHg) or a history of antihypertensive treatment. Diabetes mellitus was defined as a fasting plasma glucose ≥ 126 mg/dL or a history of hypoglycemic therapy. Smoking was defined as current smoker at the time of examination. Hypercholesterolemia was defined as a fasting serum total cholesterol ≥ 220 mg/dL or a history of statin medication. Data indicating coronary arterial occlusive disease (CAOD) was defined as a history of CAOD and percutaneous coronary interventions or coronary artery bypass grafting. Chronic kidney disease (CKD) was defined as an estimated glomerular filtration rate (eGFR) <60 mL/min/1.73 m^2^. In addition to the above-mentioned clinical data, systolic and diastolic blood pressures were also measured.

### Measurement of the NLR and Laboratory Parameters

Plasma sample data were collected for all study participants and included only tests performed within 1 month of the radiological examination. Laboratory parameters such as fasting blood glucose, total cholesterol, triglycerides, white blood cell count, neutrophil count, lymphocyte count, and eGFR were tested in the biochemistry department of the hospital. The eGFR was calculated using the abbreviated Modification of Diet in Renal Disease Study Equation (186 × serum creatinine^−1.154^ × age^−0.203^ × 0.742 [*if female*)]) (30). Estimated neutrophil and lymphocyte counts were calculated from the differential count of complete blood cell counts, which was presented as the percentage of the whole white blood cell count. The NLR was calculated as the ratio of the neutrophil count to the lymphocyte count.

### Radiological Evaluation

Brain MRI and MRA were performed using one of three 1.5-T MR systems (Sonata, Siemens Healthcare, Germany; Signa Excite, GE Healthcare, USA; SignaHDx, GE Healthcare). Image interpretation was performed by one neurologist and one radiologist, who were blinded to clinical and laboratory data. An SLI was defined as a small (3–15 mm in diameter) cavitated lesion in an area supplied by deep perforating arteries that showed low signal intensity on T1-weighted imaging (repetition time [TR]/echo time [TE] = 560/14 ms) (31). All series contained 16 axial images with a slice thickness of 7 mm and a 2-mm inter-slice gap. For analysis of cerebral WMHs, FLAIR images (TR/TE = 9000/105 ms; inversion time, 2500 ms) were used in accordance with the MR imaging protocol. The presence of cerebral WMHs was evaluated on FLAIR images and the severity of cerebral WMHs was assessed using the Fazekas scoring system (32). The severity of the periventricular WMH (PVWMH) was graded with the following scale: 0 = absent; 1 = cap- or pencil-thin lining; 2 = smooth halo; and 3 = irregular PVWMH extending into the deep white matter. The severity of the deep subcortical WMH (DSWMH) was rated with the following scale: 0 = absent; 1 = punctuate foci; 2 = beginning of confluence of foci; and 3 = large confluent areas. The presence of WMHs was defined as a total score in the PVWMH and DSWMH of 2 or more. LAA was identified as either marked stenosis (≥50%) or total occlusion of an IC or EC cerebral artery on brain MRA (MAGNETOM Symphony, Siemens, Heidelberg, Germany) (33). One neurologist and one radiologist who were unaware of patients' clinical information retrospectively analyzed the MRA data. A diagnosis of EC or IC atherosclerosis was made only when both investigators concurred. Using the method described in the Warfarin vs. Aspirin for Symptomatic Intracranial Disease study (34), IC atherosclerosis was defined as atherosclerosis in the following arteries: anterior cerebral, middle cerebral, posterior cerebral, distal internal carotid, distal vertebral, and basilar arteries. EC atherosclerosis was defined as atherosclerosis in the proximal internal carotid and vertebral arteries using the method described in the North American Symptomatic Carotid Endarterectomy Trial (35).

### Statistical Analysis

To evaluate the factors associated with the NLR, participants were categorized by tertile based on NLR level (first tertile, <1.37; second tertile, 1.37–2.06; and third tertile, more than 2.06). Baseline characteristics were compared among NLR tertiles. Continuous variables are reported as mean ± standard deviation, whereas categorical variables are reported as frequency and percentage. Analysis of variance was used to compare the continuous variables, whereas chi-square test was used to compare the categorical variables. Logistic regression analyses were performed to evaluate the factors associated with the individual radiological indices of LAA (EC atherosclerosis and IC atherosclerosis) and CSVD (WMHs and SLI). In the logistic regression analyses, NLR was treated as categorical variable (NLR tertiles) or log-transformed continuous variable (logNLR). Adjustments were performed for the following established atherosclerotic risk factors: age, sex, hypertension, diabetes, hyperlipidemia, current smoking, CAOD, and CKD. Odds ratios (ORs) and 95% confidence intervals (CIs) were calculated from the logistic regression models.

For evaluating the ability of NLR in predicting EC/IC atherosclerosis, we illustrated receiver operating characteristic (ROC) curves of univariate and multivariate logistic regression models and calculated the area under the curve (AUC). The optimal cut-off value of NLR was determined at the level with the highest Youden's index (sensitivity+specificity−1). To evaluate a goodness of fit of logistic regression models, we illustrated calibration plot presenting observed vs. predicted risk using the *val.prob* function in the ‘rms' R package (https://hbiostat.org/R/rms/). Statistical analyses were conducted using SPSS (ver. 18.0; SPSS Inc., IL, USA) and R software, version 3.6.3 (The R Foundation for Statistical Computing, Vienna, Austria; http://www.R-project.org/). A two-sided *p* < 0.05 was considered statistically significant.

## Results

The demographic characteristics of the study participants are summarized in Table 1. The clinical characteristics of the 950 participants included in this study were analyzed according to NLR tertile. There were significant differences among the NLR tertiles with regard to demographic data and vascular risk factor frequency. In univariate analysis, participants in the highest NLR tertile group tended to be older and had higher prevalence of CKD and a higher fasting glucose level than the lowest one (Table 1). The highest NLR tertile group had a lower prevalence of hyperlipidemia and lower plasma levels of cholesterol and triglyceride compared with the lowest tertile group. The white blood cell and neutrophil counts were higher and the lymphocyte counts lower in the highest NLR tertile group than in the lowest group (Table 1).

**Table 1:** Clinical characteristics of participants according to NLR tertile.

	**ALL (*N* = 950)**	**T1 (*N* = 316)**	**T2 (*N* = 317)**	**T3 (*N* = 317)**	***P***
Sex (female)	603 (63.5)	212 (67.1)	196 (61.8)	195 (61.5)	0.262
Age, years	65.6 ± 8.7	63.7 ± 8.1	65.3 ± 8.4	67.7 ± 9.2	<0.001
Hypertension	540 (56.8)	167 (52.8)	182 (57.4)	191 (60.3)	0.165
Diabetes mellitus	211 (22.2)	64 (20.3)	68 (21.5)	79 (24.9)	0.341
Hyperlipidemia	298 (31.4)	110 (34.8)	107 (33.8)	81 (25.6)	0.023
Current smoking	201 (21.2)	65 (20.6)	66 (20.8)	70 (22.1)	0.883
CAOD	51 (5.4)	15 (4.7)	12 (3.8)	24 (7.6)	0.089
CKD	189 (20.0)	43 (13.6)	66 (20.9)	80 (25.5)	0.001
Statin medication	216 (22.7)	78 (24.7)	78 (24.6)	60 (18.9)	0.140
SBP, mmHg	132.5 ± 19.1	131.3 ± 17.7	132.6 ± 19.4	133.5 ± 20.1	0.341
DBP, mmHg	80.6 ± 11.8	80.4 ± 11.4	80.6 ± 12.2	80.8 ± 11.7	0.906
Fasting glucose, mg/dL	124.4 ± 47.7	119.5 ± 41.8	119.7 ± 38.3	134.0 ± 58.4	<0.001
eGFR, mL/min/1.73 m^2^	73.9 ± 18.2	76.4 ± 16.2	73.8 ± 17.9	71.6 ± 20.1	0.004
Total cholesterol, mol/U	5.0 ± 1.1	5.1 ± 1.0	5.0 ± 1.0	4.9 ± 1.3	0.023
Triglyceride, mol/U	1.7 ± 1.1	1.8 ± 1.2	1.6 ± 0.9	1.6 ± 1.1	0.044
White blood cell count, × 10^9^/L	6.7 ± 2.2	5.9 ± 1.7	6.5 ± 1.6	7.7 ± 2.6	<0.001
Neutrophil count, × 10^9^/L	3.9 ± 1.9	2.7 ± 0.9	3.7 ± 1.0	5.4 ± 2.2	<0.001
Lymphocyte count, × 10^9^/L	2.1 ± 0.8	2.6 ± 0.8	2.2 ± 0.6	1.6 ± 0.6	<0.001

*T1, lowest tertile; T2, middle tertile; T3, highest tertile; NLR, neutrophil/lymphocyte ratio; CAOD, coronary arterial occlusive disease; CKD, chronic kidney disease; SBP, systolic blood pressure; DBP, diastolic blood pressure; eGFR, estimated glomerular filtration rate*.

**Figure 1:**
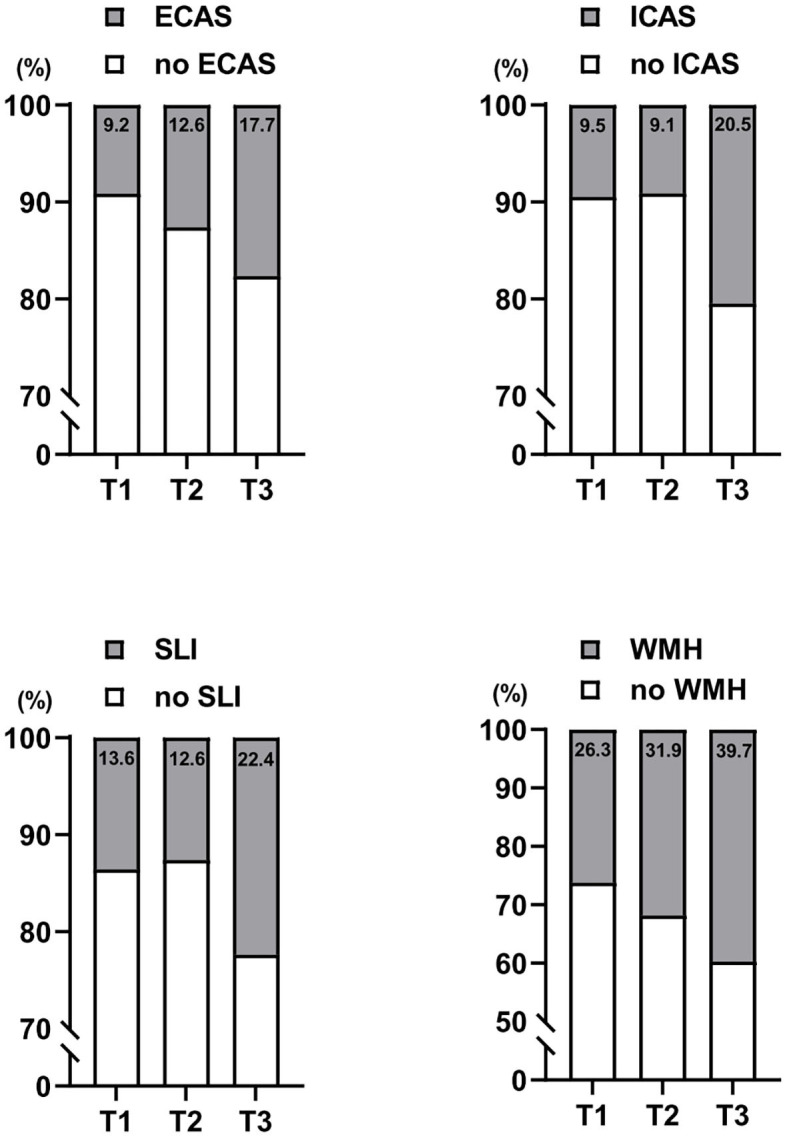
Distributions of extracranial (EC) atherosclerosis, intracranial (IC) atherosclerosis, silent lacunar infarction (SLI), and cerebral white matter hyperintensities (WMHs) by serum neutrophil/lymphocyte ratio (NLR) levels categorized in tertiles. T1, lowest tertile; T2, middle tertile; T3, highest tertile.

In radiological examinations, EC and IC atherosclerosis were found in 13.2 and 13.1% of study participants, respectively. The prevalences of EC and IC atherosclerosis were significantly different among the three NLR tertile groups, with a higher prevalence of EC and IC atherosclerosis in the highest NLR tertile group than in the lowest group (Table 2 and Figure 1). SLI and WMHs were found in 16.2% and 32.6% of our study participants, respectively. The prevalences of SLI and WMHs were significantly different among the three NLR tertile groups, with higher prevalences of SLI and WMHs in the highest NLR tertile group than in the lowest group (Table 2 and Figure 1).

**Table 2:** Prevalences of EC atherosclerosis, IC atherosclerosis, SLI, and WMHs in study participants according to NLR tertile.

	**ALL (*N* = 950)**	**T1 (*N* = 316)**	**T2 (*N* = 317)**	**T3 (*N* = 317)**	***P***
EC atherosclerosis	125 (13.2)	29 (9.2)	40 (12.6)	56 (17.7)	0.006
IC atherosclerosis	124 (13.1)	30 (9.5)	29 (9.1)	65 (20.5)	<0.001
SLI	154 (16.2)	43 (13.6)	40 (12.6)	71 (22.4)	0.001
WMH	310 (32.6)	83 (26.3)	101 (31.9)	126 (39.7)	0.001

*T1, lowest tertile; T2, middle tertile; T3, highest tertile; NLR, neutrophil/lymphocyte ratio; EC, extracranial; IC, intracranial; SLI, silent lacunar infarction; WMHs, cerebral white matter hyperintensities*.

We next conducted logistic regression analyses among NLR tertile groups to determine whether NLR is independently associated with the presence of EC atherosclerosis, IC atherosclerosis, SLI, and WMHs. After adjustment for cardiovascular risk factor variables, participants in the highest NLR tertile had more EC atherosclerosis (OR: 1.88; 95% CI: 1.14–3.09) than participants in the lowest tertile (Table 3). Participants in the highest NLR tertile also had more IC atherosclerosis (OR: 1.87; 95% CI; 1.15–3.06) than participants in the lowest tertile (Table 3) after adjustment for cardiovascular risk factor variables. The prevalence of SLI and CSVD were not different across three NLR tertile groups in multivariable analysis. Table 4 shows the results of multivariate logistic regression analysis with log-transformed continuous NLR (logNLR) values instead of NLR tertile groups. As in the previous analysis with tertiles, high level of logNLR were significantly associated with the presence of EC atherosclerosis (OR: 1.58; 95% CI: 1.13–2.19) and IC atherosclerosis (OR: 1.44; 95% CI: 1.02–2.03).

**Table 3:** Logistic regression analysis for EC atherosclerosis, IC atherosclerosis, SLI, and WMHs according to NLR tertile.

	**EC atherosclerosis**	***P***	**IC atherosclerosis**	***P***	**SLI**	***P***	**WMH**	***P***
	**OR (95% CIs)**		**OR (95% CIs)**		**OR (95% CIs)**		**OR (95% CIs)**	
T1	Ref	-	Ref	-	Ref	-	Ref	-
T2	1.29 (0.76–2.18)	0.343	0.81 (0.46–1.42)	0.463	0.71 (0.43–1.16)	0.169	1.12 (0.77–1.63)	0.566
T3	1.88 (1.14–3.09)	0.014	1.87 (1.15–3.06)	0.012	1.23 (0.78–1.93)	0.379	1.29 (0.89–1.88)	0.176

*Data were adjusted by age, sex, hypertension, diabetes, hyperlipidemia, current smoking, coronary arterial occlusive disease, and chronic kidney disease. T1, lowest tertile; T2, middle tertile; T3, highest tertile; Ref, reference group; EC, extracranial; IC, intracranial; SLI, silent lacunar infarction; WMHs, cerebral white matter hyperintensities; NLR, neutrophil/lymphocyte ratio; OR, odds ratio; CIs, confidence intervals*.

**Table 4:** Logistic regression analysis with logNLR for EC atherosclerosis, IC atherosclerosis, SLI, and WMHs.

	**EC atherosclerosis**	***P***	**IC atherosclerosis**	***P***	**SLI**	***P***	**WMH**	***P***
	**OR (95% CIs)**		**OR (95% CIs)**		**OR (95% CIs)**		**OR (95% CIs)**	
LogNLR	1.58 (1.13–2.19)	0.007	1.44 (1.02–2.03)	0.035	1.15 (0.82–1.59)	0.412	1.20 (0.91–1.59)	0.188

*Data were derived from logistic regression models adjusted by age, sex, hypertension, diabetes, hyperlipidemia, current smoking, coronary arterial occlusive disease, and chronic kidney disease. EC, extracranial; IC, intracranial; SLI, silent lacunar infarction; WMHs, cerebral white matter hyperintensities; LogNLR, natural log-transformed neutrophil/lymphocyte ratio; OR, odds ratio; CIs, confidence intervals*.

**Figure 2:**
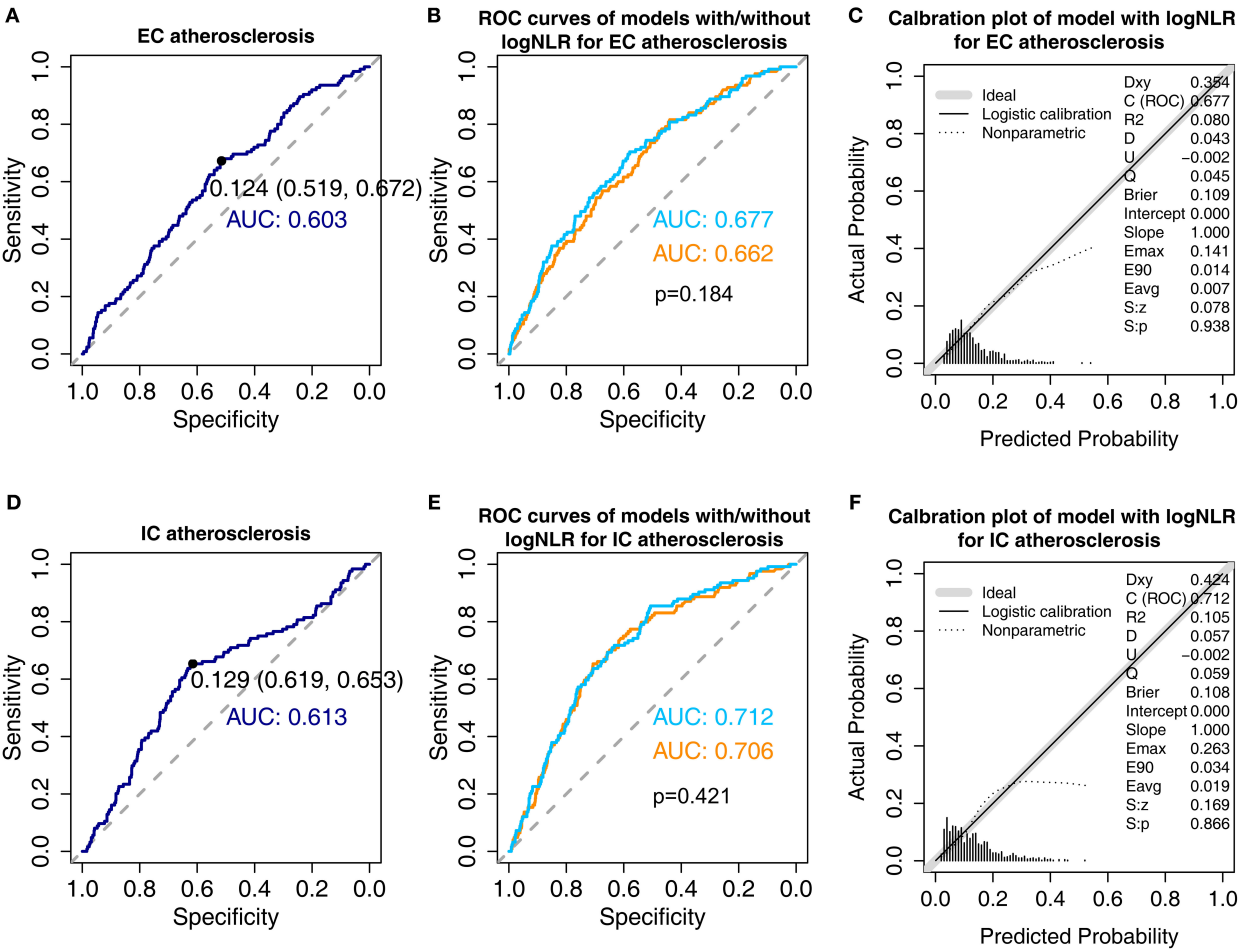
Receiver operative characteristic curves and calibration plots of the models for extracranial (EC) atherosclerosis and intracranial (IC) atherosclerosis. **(A)** Receiver operative characteristic (ROC) curve of univariate logistic regression model for EC atherosclerosis with natural log-transformed serum neutrophil/lymphocyte ratio (logNLR). The black point shows optimal cut-off point of logNLR derived by Youden's method. The numbers in parentheses indicate specificity and sensitivity at the cut-off point **(B)** Comparison of area under curve (AUC) for EC atherosclerosis in the multivariate models with and without logNLR. Skyblue indicates AUC of the model with logNLR and orange indicates AUC of the model without logNLR. Adjustments were performed for the variables listed in Table 4. **(C)** Calibration plot for multivariate logistic regression with logNLR for EC atherosclerosis. **(D)** ROC curve of univariate logistic regression model for IC atherosclerosis with logNLR. **(E)** Comparison of AUC for IC atherosclerosis in the multivariate models with and without logNLR. **(F)** Calibration plot of the multivariate logistic regression with logNLR for IC atherosclerosis.

We constructed ROC curves to evaluate optimal cut-off value and predictability of NLR (Figure 2). In the ROC curves based on univariate logistic regression model with logNLR, optimal cut-off of logNLR were 0.124 (specificity: 0.519; sensitivity: 0.672) for EC atherosclerosis and 0.129 (specificity: 0.619, sensitivity: 0.653) for IC atherosclerosis (Figures 2A,D). AUC for EC atherosclerosis and IC atherosclerosis were 0.603, and 0.613, respectively. We evaluated AUC of multivariate logistic models for EC atherosclerosis and IC atherosclerosis by adding logNLR (Figures 2B,E). Addition of logNLR to the models increased AUC, but it did not reach statistical significance. Figures 2C,F show calibration plots of the multivariate logistic regression models with logNLR predicting the EC atherosclerosis and IC atherosclerosis.

## Discussion

In the present study, participants in the highest tertile had a higher prevalence of radiological indices of both LAA and CSVD. The principal finding of our study is that the NLR is significantly associated with indices of LAA (EC and IC atherosclerosis). The statistical significance remained after adjustment for cardiovascular risk factors. These results are consistent with previous findings indicating that the NLR is strongly correlated with and precisely predicts cerebral atherosclerosis (36, 37). Meng et al. (37) found that the NLR is positively associated with carotid intima-media thickness in a rabbit model administered a high-fat diet. Another study by Demirkol et al. showed that the NLR is related to carotid intima-media thickness in patients with CAOD and cardiac syndrome X (36). In this study, participants with EC or IC atherosclerosis showed a high NLR due to a significant increase in neutrophils and decrease in lymphocytes. Neutrophils usually accumulate on the vascular wall, causing vascular inflammation. This can lead to endothelial dysfunction and subsequent atherosclerosis by reducing the use of nitrogen oxide (37, 38). Furthermore, neutrophils infiltrate human carotid atherosclerotic plaques, playing roles in the destabilization and subsequent rupture of the atherosclerotic plaque (39, 40). The exact role of lymphocytes in atherosclerosis is still unclear. A recent study suggested that some lymphocytes, such as regulatory T cells, participate in the healing process in inflammatory milieu by secreting IL-10 (41). Therefore, a decreased lymphocyte count may accelerate atherosclerosis. Overall, our study demonstrated that the NLR is a useful indicator of the development of atherosclerosis in EC or IC arteries, even in asymptomatic individuals.

We also examined whether NLR is differentially associated with EC and IC atherosclerosis among the LAA. The reason for this classification is due to differences in morphological, metabolic and biological properties between the IC and EC arteries. IC arteries, compared to similar sized EC arteries, are muscular arteries containing a small number of medial elastic fibers, a thick and dense inner elastic layer, and a few adventitial vasa vasorum, lacking an external elastic lamina (42–44). These results suggest that the greater the activity of antioxidant enzymes in IC arteries compared to other arteries of similar size, the greater the resistance to atherosclerosis to contribute to a lower level of atherosclerosis. Thus, we analyzed EC and IC atherosclerosis separately for these reasons.

According to the ROC curves, the optimal cut-off values of logNLR were 0.124 for EC atherosclerosis and 0.129 for IC atherosclerosis which were equal to 1.33 and 1.35 for NLR raw value. Although, there were no definite cut-off values of NLR to predict atherosclerosis, recent meta-analysis suggested the NLR cut-off values ranged from 1.20 to 3.67 to predict cardiovascular diseases including acute coronary syndrome, stroke, and peripheral artery disease (45).

The second major finding of our study is the lack of an association between the NLR and indices of CSVD (SLI and WMHs). Previous results regarding the association between the NLR and CSVD are also controversial. One study showed that the NLR is associated with cerebral WMHs in 2,875 healthy Korean participants (46), whereas another study did not find any association between them (47). This discrepancy is attributable to differences in patient characteristics, methodologies, and definitions of LAA and CSVD among studies. Although systemic inflammation is one of the major pathogenic processes leading to atherosclerosis, it is still unclear whether systemic inflammation is associated with CSVD. Several reports demonstrated that inflammation also plays a role in the development of CSVD (18–20), whereas others did not (13). Georgakis et al. (13) found that genetically determined circulating MCP-1 was associated with LAA, unlike MCP-1 levels, and that other cytokines were not associated with the small-vessel stroke type, even though the sample size was larger than for other stroke subtypes. LAA had a stronger activation of inflammation than CSVD in terms of stroke pathogenesis, which has been reported to be associated with changes in CRP levels (48). These findings suggest that inflammatory processes may be less important in CVSD than in LAA. Further study is required to clarify the role of NLR as an indicator of the development of CSVD.

The present study has the following limitations. First, because this study was retrospective in design, a selection bias existed. Furthermore, the demographic characteristics were not the same as those of the general population because the study participants visited the outpatient department due to underlying cardiovascular risk factors. Second, because all study participants were Koreans, the results cannot be generalized to other ethnicities. The results may differ across ethnicities because the prevalence of IC atherosclerosis and CSVD is higher in Asians than in Western populations. Third, the definition in the present study of atherosclerosis based on brain MRA did not include mild cases due to low MRA resolution. Furthermore, LAA is commonly comorbid with CSVD. This suggests that CSVD may also be related to the NLR. Therefore, the possibility that the over-interpretation of this study results occurred cannot be completely excluded. We did not consider the LAA-only (10.2%) and CSVD-only (25.7%) groups respectively because of small proportion of patients with LAA-only, but an investigation of the association between LAA-only, mixed type (12.4%) and NLR might increase the power of the predictions of the study. Fourth, the NLR may also be affected by diseases other than cerebral vascular disease, such as other inflammatory conditions, including diabetes mellitus, hypertension, auto-inflammatory diseases, and malignancies (22–26). In this study, although patients with positive history and sign of recent infections were excluded, patients with autoimmune disease, malignancy, or any other inflammatory disorders were not completely excluded. And, the relationship with another widely used inflammation marker, hs-CRP, had not been analyzed. Nevertheless, this study found a specific association between NLR and LAA, which may be meaningful. Fifth, the NLR is reversely associated with serum cholesterol and triglyceride. The exact mechanism of these results remains unknown. One explanation is NLR is associated with the variability of cholesterol, rather than baseline cholesterol level (49). Further study is required to clarify the association between hyperlipidemia and NLR. Finally, it is uncertain whether the NLR is a risk factor or a consequence of cerebral atherosclerosis. A firm conclusion could not be drawn based on the results of the present study. Thus, further prospective observations conducted in the general population are necessary to validate our results.

## Conclusions

The NLR was independently associated with indices of LAA in neurologically normal Koreans. Conversely, the NLR did not show any significant value for the presence of CSVDs such as SLI and WMHs. Our study provides evidence of a possible role of inflammation in the atherosclerosis of EC and IC arteries but not in microvascular pathologies in the brain. The participants with high NLR in our study, although having no history of stroke, are in subclinical inflammatory conditions, which would lead to vascular injury and subsequent atherosclerosis. Therefore, we suggest that the NLR serves as an indicator of LAA even in asymptomatic people. Although further studies are needed, treatments that reduce inflammation are necessary to prevent the progression of cerebral atherosclerosis and subsequent stroke.

## Data Availability Statement

The raw data supporting the conclusions of this article will be made available by the authors, without undue reservation.

## Ethics Statement

The studies involving human participants were reviewed and approved by Institutional Ethical Committee of the CHA Bundang Medical Center (IRB approval no. 2010-083). The patients/participants provided their written informed consent to participate in this study.

## Author Contributions

DC and KOL: conception, design, drafting of manuscript, acquisition of data, and final approval of manuscript. J-WC, NK, and S-HK: design, acquisition of data and statistical analyses, revision of manuscript, and final approval of manuscript. O-JK: design, acquisition of data, revision of manuscript, and final approval of manuscript. S-HO and WK: conception, design, analysis and interpretation of data, drafting of the manuscript, revision of manuscript, and final approval of manuscript. All authors contributed to the article and approved the submitted version.

## Conflict of Interest

The authors declare that the research was conducted in the absence of any commercial or financial relationships that could be construed as a potential conflict of interest.
